# Mortality among Patients with Cleared Hepatitis C Virus Infection Compared to the General Population: A Danish Nationwide Cohort Study

**DOI:** 10.1371/journal.pone.0022476

**Published:** 2011-07-18

**Authors:** Lars Haukali Omland, Peer Brehm Christensen, Henrik Krarup, Peter Jepsen, Nina Weis, Henrik Toft Sørensen, Niels Obel, on behalf of the DANVIR Cohort Study

**Affiliations:** 1 Department of Infectious Diseases, Copenhagen University Hospital, Rigshospitalet, Copenhagen, Denmark; 2 Department of Infectious Diseases, Odense University Hospital, Odense, Denmark; 3 Department of Clinical Biochemistry, Aarhus University Hospital, Aalborg Hospital, Aalborg, Denmark; 4 Department of Clinical Epidemiology, Aarhus University Hospital, Aarhus, Denmark; 5 Department of Medicine V (Hepatology and Gastroenterology), Aarhus University Hospital, Aarhus, Denmark; 6 Department of Infectious Diseases, Copenhagen University Hospital, Hvidovre Hospital, Copenhagen, Denmark; 7 Department of Epidemiology, Boston University School of Public Health, Boston, Massachusetts, United States of America; Hannover Medical School, Germany

## Abstract

**Background:**

The increased mortality in HCV-infected individuals partly stems from viral damage to the liver and partly from risk-taking behaviours. We examined mortality in patients who cleared their HCV-infection, comparing it to that of the general population. We also addressed the question whether prognosis differed according to age, substance abuse (alcohol abuse and injection drug use) and comorbidity.

**Methodology/Principal Findings:**

Patients with cleared HCV-infection were categorized into one of 8 groups according to age (20–39 years or 40–69 years) and patient characteristics (no substance abuse/no comorbidity; substance abuse/no comorbidity; no substance abuse/comorbidity; and substance abuse/comorbidity). For each patient, 4 age- and gender-matched individuals without substance abuse or comorbidity were selected from the general population, comprising a total of 8 comparison cohorts. We analyzed 10-year survival and used stratified Cox Regression analysis to compute mortality rate ratios (MRRs), comparing mortality between the 8 patient groups and the comparison cohorts, adjusting for personal income. Among patients without substance abuse or comorbidity, those aged 40–69 years had the same mortality as the comparison cohort (10-year survival: 95% (95% confidence interval [CI]: 93%–97%), MRR: 1.3 (95% CI: 0.8–2.3)), whereas those aged 20–39 years had higher mortality than the comparison cohort (10-year survival: 93% versus 99%, MRR: 5.7 (95% CI: 2.3–14.0). For both age categories, substance abuse and comorbidity decreased survival and increased MRRs. Patients aged 40–69 years with substance abuse and comorbidity suffered from substantial mortality (MRR: 12.5 (95% CI: 5.1–30.6)).

**Conclusions:**

Mortality in patients aged 40–69 years with cleared HCV-infection is comparable to individuals without HCV, provided they have no substance abuse or comorbidity. Any substance abuse and/or comorbidity not captured in the registries used for our study could explain the increased mortality in patients aged 20–39 years without documented substance abuse or comorbidity.

## Introduction

Large cohort studies have documented 2- to 18-fold higher mortality among HCV-infected patients compared to the general population [1]–[4]. Increasingly effective treatments provide optimism about improved life expectancy in HCV-infected patients [5], [6]. However, they are at elevated risk of both liver-related death and of unnatural causes of death/drug-related death [1], [3], attributable to a high prevalence of lifestyle-related risk factors (alcohol abuse and injection drug use (IDU)) [3]. Thus, HCV-infected patients may remain at increased risk of death even after clearing the virus. Estimates of absolute mortality risk are needed for these patients, disaggregated by presence or absence of comorbidity, alcohol abuse and IDU. To date such estimates have not been reported.

We therefore conducted a nationwide cohort study of patients with cleared HCV-infection (without HIV), estimating 5- and 10-year risks of death compared to that of an age- and gender matched comparison cohort without HIV, comorbidity or substance abuse (alcohol abuse or IDU).

Patients were categorized into one of 8 groups according to age (20–39 years or 40–69 years) and characteristics at time of diagnosis, *i.e.*, those with no substance abuse or comorbidity; those with substance abuse; those with comorbidity; and those with substance abuse and comorbidity. Five- and 10-year mortality in each of these subgroups was estimated and compared to that of corresponding subgroups in the comparison cohort.

## Materials and Methods

### Setting

Denmark has a population of 5.5 million persons [7] and an estimated population of chronically HCV-infected patients of 15.000 (prevalence of 0.3) [8]. Medical care, including antiviral treatment, is provided free of charge to all HCV-infected residents. It has been estimated that only 2% of this patient population has been treated with interferon [9]. Suboptimal screening activity and ineffective prevention and management strategies could explain this rather low percentage [9]. However, for HCV-infected patients referred for evaluation in one of the 15 centres specialised in treating viral hepatitis in Denmark, the 5-year probability of being treated with interferon is 33% (95% confidence interval (CI): 28%–38%) [10].

### Data sources

We used the unique 10-digit civil registration number assigned to all individuals in Denmark to link individual-level data from the following data sources [11]:

#### Danish HCV cohort (DANVIR)

HCV RNA negative patients were identified from the DANVIR cohort, a prospective research cohort consisting of all patients tested for hepatitis B or HCV-infection in 14 of the 18 Danish laboratories that perform such testing [3], [12], [13]. In total, 13,005 patients are registered in the DANVIR cohort during the period between 1^st^ January 1991 and 1^st^ January 2007. The number under observation has increased during the study period from 781 at 1^st^ January 1995, 4139 at 1^st^ January 2000 and 9789 at 1^st^ January 2005 (0.2% of the Danish population). Enrolments increased from approximately 200/year the first 5 years until it peaked in 2004 (1807 enrolments). Patients are included the first date of a test indicating HCV-exposure (a positive test for HCV-antibodies and/or HCV RNA). Many, but not all patients have both a positive test for HCV antibodies and an available test for HCV RNA, enabling a determination of whether or not they have cleared their HCV-infection [13]. Results of genotype test are available only for few patients.

#### The Danish National Hospital Registry (DNHR)

The DNHR, established in 1977, records all hospital admissions to non-psychiatric hospitals in Denmark. Data from outpatient and emergency department visits were added starting in 1995. Registry records for each medical contact include dates of admission and discharge and up to 20 discharge diagnoses, coded according to the *International Classification of Diseases*, 8th revision (ICD-8) through 1993 and 10th revision (ICD-10) from 1994 onward. Diagnostic codes are assigned by physicians [14]. We used this registry to ascertain HIV status, alcohol abuse and IDU. In 2005, DNHR recorded 1,205,000 discharges and 6,336,000 outpatient visits [15]. Of the 13,005 patients in DANVIR, 12,150 patients (93%) had one or more contact registered in DNHR before, on or after the date of inclusion in DANVIR.

#### The Registry of Drug Abusers Undergoing Treatment (RDT)

The RDT, run by the National Board of Health, has registered all individuals in Denmark receiving treatment for drug addiction since 1996 [16]. Treatment of drug addiction in Denmark is restricted to referral centers, which must supply data to the RDT in order to obtain funding. The RDT registers the main drug used (opioids, hashish or centrally acting drugs), whether patients are treated as outpatients or inpatients and whether patients receive substitution therapy or not. In 2006, 13.441 patients received treatment and were registered in RDT with 5426 initiating treatment in 2006 and the remainder (8015) initiating treatment before 2006. Of the 5426 initiating treatment in 2006, 1329 had not previously been treated. Of the 13.005 patients in DANVIR, 6143 (47%) were registered in RDT before, on or after the date of inclusion in DANVIR [17]. We also ascertained IDU from this registry.

#### The Danish Civil Registration System (CRS)

The CRS, established in 1968, updates the vital status of all Danish citizens on a daily basis, including the date of death or emigration. The CRS also stores information on municipality of residence and migration for all Danish residents [11]. As of November 2005, the CRS included information on 8.2 million persons, of which 5.4 million were alive and living in Denmark. The cornerstone of CRS's extreme completeness in terms of vital status (including emi- and immigration) is the civil registration number used by all national registries as well as Danish legislation, according to which all residents in Denmark are obliged to inform the authorities about any change of permanent address within 5 days [11], [18], [19]. Failure to supply this information will results in loss of all of benefits provided by the state - therefore it is very unlikely that this mandatory information is not reported [19].

#### The Integrated Database for Labour Market Research (IDA)

The IDA, established by Statistics Denmark in 1980, records individual-level data on the annual personal income of all Danish citizens [20].

#### Danish Registry of Causes of Death (DRCD)

The DRCD contains information extracted from all Danish death certificates since 1943 [21]. Whenever a Danish citizen dies, the attending physician must report the cause of death. Up to 4 diagnoses can be specified to describe the chain of events leading to death. Causes of deaths occurring during the study period were coded according to ICD-10.

### Study population

We focused on patients in DANVIR, who were a) diagnosed with a positive test for HCV antibodies and who subsequently tested negative for HCV RNA (the date of the latter test was used as the index date), b) age 20 years or older on the index date and c) not coinfected with HIV. Patients from DANVIR fulfilling these criteria from 1 January 1996 to 31 December 2006 were included in the study. In this way, 2320 of the 13005 patients registered in DANVIR (18%) were included in the present study. For each of these 2320 patient, 4 age- and gender-matched individuals without HCV-infection were identified from the CRS (N = 9280). These individuals, who served as the comparison cohort, had to be free of HIV infection, alcohol abuse, IDU and comorbidity, as defined below.

### Information on study subjects

*Comorbidity* was measured based on the Charlson Comorbidity Index (CCI) score derived from diagnoses registered in the DNRP before the index date [22], [23]. The intention with the creation of the CCI was to develop a prognostic score based on comorbid conditions, which could predict (short term) mortality in longitudinal studies [22]. The score has been validated a number of times [23]–[25] and has proved capable of also predicting long-term mortality [22]. The CCI assigns a score between 1 and 6 to a range of diseases, with the sum of individual scores serving as a measure of patients' overall comorbidity. We identified comorbid diseases using the ICD-10 codes provided by Quan *et al.* [25] (matching ICD-8 codes to ICD-10 codes as closely as possible [Appendix 3]) and categorized patients as having comorbidity or not (yes/no). Liver diseases were excluded from the CCI for the purposes of the present study (Appendix 3).

*Alcohol abuse*, *IDU* and *HIV status* were ascertained from the DNRP and RDT, as specified in the Appendix 1 and previously described [3]. We defined *substance abuse* as having a diagnosis of alcohol abuse and/or IDU as of the index date.

*Yearly income* was ascertained for the calendar year preceding HCV RNA assessment, characterized as 0%–49%, 50%–99% or 100+% of the average income for all Danish citizens of the same age and gender in the same calendar year.

#### Cause of death

Based on the diagnosis listed as the primary cause of death, we categorized deaths into one of 3 categories: liver-related deaths, non-liver-related natural deaths or unnatural deaths (see Appendix 2 for details).

### Statistical analysis

Study outcomes were time to all-cause mortality and time to cause-specific mortality. Time at risk was calculated from the index date until death, emigration or 31 December 2006, whichever came first. We analyzed individuals aged 20–39 years and 40–69 years separately, as younger HCV-infected patients are at particularly high risk of death compared to the general population [3]. Mortality was analyzed using Kaplan-Meier survival analyses. We used stratified Cox regression models (according to the matched design) to analyze whether patients with cleared HCV-infection had higher mortality than the comparison cohort. We then reran the Cox regression models with adjustment for personal income, categorized as 0%–49%, 50%–99% and 100+% of national average income. We analyzed cause-specific mortality in the same way as for overall mortality, but without adjusting for income (due to the small number of events in each category).

We then further subdivided the 2 subgroups of patients with cleared HCV-infection (*i.e.*, those aged 20–39 years and those aged 40–69 years) according to their characteristics (patients with no comorbidity or substance abuse; patients with substance abuse; patients with comorbidity; and patients with comorbidity and substance abuse), resulting in a total of 8 subgroups of patients with cleared HCV-infection. The analyses of overall mortality were then repeated for each of these subgroups in order to identify patients with a particularly good or bad prognosis.

### Ethics

Since the study is entirely based on data from national registries and clinical databases, there was according to Danish law no request for an ethical permission (Denmark has no Institutional review boards). The study was approved by Danish Data Protection Agency.

## Results

### Descriptive data

From the DANVIR cohort we identified 2,320 patients with cleared HCV-infection. The median age was 41 years at study inclusion. Characteristics of these patients and the comparison cohort are provided in Table 1. 47% of the patients were IDU – with more IDUs among the younger than among the older patients. 11% were alcohol abusers (current or former) and 16% had comorbidity. There were more patients with alcohol abuse and comorbidity among older patients than among the younger patients. Further, among those patients with comorbidity, the level of comorbidity was higher among older than younger patients. Although not included in our definition of comorbidity, more patients aged 40–69 years than patients aged 20–39 years had liver disease according to the CCI. Income was low in both the younger and the older age categories with few individuals having an income above national average for same sex and gender.

**Table 1:** Characteristics of the patients with cleared HCV-infection and the comparison cohort.

	Total cohort		20–39 years at study inclusion		40–69 years at study inclusion	
	Cleared HCV cohort	Comparison cohort	Cleared HCV cohort	Comparison cohort	Cleared HCV cohort	Comparison cohort
N	2320	9280	1067	4268	1253	5012
Male, n (%)	1303 (56)	5212 (56)	602 (56)	2408 (56)	701 (56)	2804 (56)
Age, median (interquartile range)	41 (33–51)	41 (33–51)	32 (28–36)	32 (28–36)	50 (45–57)	50 (45–57)
Year of study inclusion, median (interquartile range)	2002 (1999–2004)	2002 (1999–2004)	2001 (1999–2003)	2001 (1999–2003)	2002 (2000–2004)	2002 (2000–2004)
IDU, n (%)	1101 (47)	0 (0)	585 (55)	0 (0)	516 (41)	0 (0)
Alcohol abuse	266 (11)	0 (0)	77 (7)	0 (0)	189 (15)	0 (0)
CCI, n (%)						
0	1956 (84)	0 (0)	974 (91)	0 (0)	982 (78)	0 (0)
1–2	315 (14)	0 (0)	85 (8)	0 (0)	230 (18)	0 (0)
3+	49 (2)	0 (0)	8 (1)****	0 (0)	41 (3)*****	0 (0)
Comorbidities in CCI*						
Myocardial infarction	5 (0)	0 (0)	1 (0)	0 (0)	4 (0)	0 (0)
Congestive heart failure	8 (0)	0 (0)	0 (0)	0 (0)	8 (1)	0 (0)
Peripheral vascular disease	43 (2)	0 (0)	8 (1)	0 (0)	35 (3)	0 (0)
Cerebrovascular disease	26 (1)	0 (0)	9 (1)	0 (0)	17 (1)	0 (0)
Dementia	10 (0)	0 (0)	7 (1)	0 (0)	3 (0)	0 (0)
Chronic pulmonary disease	108 (5)	0 (0)	32 (3)	0 (0)	76 (6)	0 (0)
Connective tissue disease	31 (1)	0 (0)	3 (0)	0 (0)	28 (2)	0 (0)
Ulcer disease	79 (3)	0 (0)	12 (1)	0 (0)	67 (5)	0 (0)
Diabetes mellitus type 1 and 2	31 (1)	0 (0)	6 (1)	0 (0)	25 (2)	0 (0)
Hemiplegia	4 (0)	0 (0)	1 (0)	0 (0)	3 (0)	0 (0)
Moderate to severe renal disease	52 (2)	0 (0)	16 (1)	0 (0)	36 (3)	0 (0)
Diabetes mellitus type 1 and 2 with end organ damage	18 (1)	0 (0)	2 (0)	0 (0)	16 (1)	0 (0)
Any tumor	36 (2)	0 (0)	7 (1)	0 (0)	29 (2)	0 (0)
Leukemia	2 (0)	0 (0)	0 (0)	0 (0)	2 (0)	0 (0)
Lymphoma	10 (0)	0 (0)	4 (0)	0 (0)	6 (0)	0 (0)
Metastatic solid tumor	6 (0)	0 (0)	0 (0)	0 (0)	6 (0)	0 (0)
AIDS**	0 (0)	0 (0)	0 (0)	0 (0)	0 (0)	0 (0)
Mild liver disease***	316 (14)	0 (0)	100 (9)	0 (0)	216 (17)	0 (0)
Moderate to severe liver disease***	51 (2)	0 (0)	13 (1)	0 (0)	38 (3)	0 (0)
Income (% of national average for the same age and gender), n (%)						
0–49	1,020 (44)	1,343 (15)	553 (52)	857 (20)	467 (37)	486 (10)
50–99	889 (38)	4,028 (43)	371 (35)	1840 (43)	518 (41)	2188 (44)
100+	441 (18)	3,909 (42)	143 (13)	1571 (37)	268 (21)	2338 (47)

*: patients could have more than one category of comorbidity, and therefore the sum of the comorbidity-categories is bigger than the sum of patients with comorbidity.

**: Patients with HIV were excluded from the study.

***: The liver diseases of the CCI were not included in our comorbidity score, as they were considered being part of the causal pathway of mortality.

****: 9% ( = 8/(8+85)) of 20–39 year old patients with comorbidity.

*****: 15% ( = 41/(41−230)) of 40–69 year old patients with comorbidity.

### Mortality

**Figure 1:**
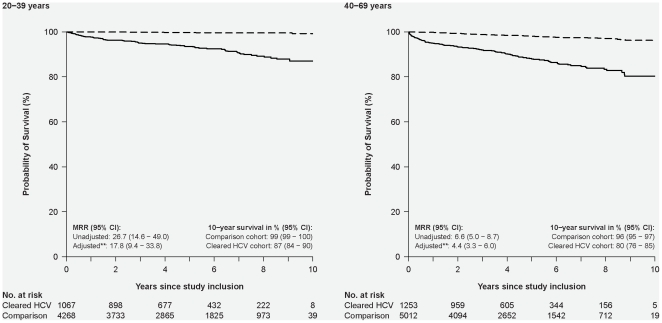
Kaplan-Meier survival plot illustrating cumulative survival in patients with cleared HCV-infection and in an age- and gender-matched comparison cohort without comorbidity, alcohol abuse or IDU, according to age at study inclusion. Solid line: Patients with cleared HCV-infection; broken line: Individuals from the comparison cohort. The left-hand graph illustrates survival among individuals aged 20–39 years at study inclusion. The right-hand graph illustrates survival in those aged 40–69 years at study inclusion.

**Figure 2:**
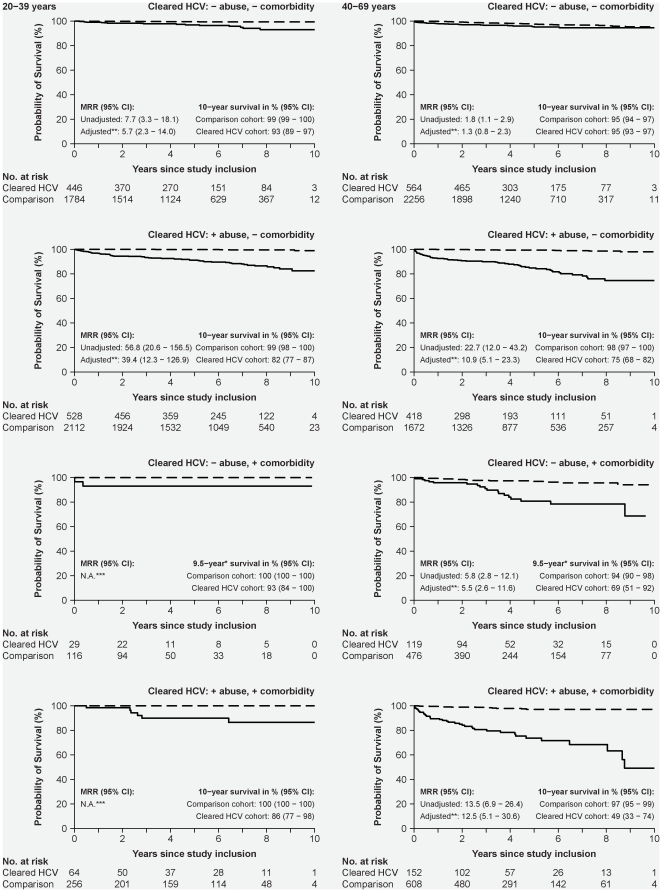
Kaplan-Meier survival plots illustrating cumulative survival in patients with cleared HCV-infection and in an age- and gender-matched comparison cohort without comorbidity, alcohol abuse or IDU, according to patient characteristics and age at study inclusion. Solid line: Patients with cleared HCV-infection; broken line: Individuals from the comparison cohort. The left-hand graphs illustrate survival in individuals aged 20–39 years at study inclusion; the right-hand graphs illustrate survival in those aged 40–69 years at inclusion. Top row: Patients with no substance abuse (alcohol abuse or IDU) or comorbidity; second row: patients with substance abuse; third row: patients with comorbidity; and last row: patients with substance abuse and comorbidity.

Patients with cleared HCV-infection had higher mortality than the comparison cohort; 10-year survival was 87% (95% CI: 84–90%) in patients aged 20–39 and 80% (95% CI: 76–85%) in those aged 40–69 years (Figure 1). Patients with substance abuse (alcohol abuse or IDU) were at particularly high risk of death in both age categories (Figure 2). Patients aged 40–69 years at study inclusion with comorbidity also had substantially shorter survival (10-year survival: 93% (95% CI: 89–97%)) than the comparison cohort (adjusted MRR = 5.5 (95% CI: 2.6–11.6)). The number of younger patients with comorbidity and no substance abuse was so small that no definite conclusions could be drawn. Presence of both comorbidity and substance abuse was associated with a particularly sinister prognosis among those aged 40–69 years, with an estimated 10 year survival of 49% (95% CI: 22%–74%). Importantly, mortality in patients aged 40–69 years without comorbidity or substance abuse was comparable to that of the comparison cohort, whereas younger patients without comorbidity or substance abuse still had substantially higher mortality than the comparison cohort (10-year survival: 93% versus 99%; adjusted MRR = 5.7 (95% CI: 2.3–14.0).

### Cause-specific mortality

**Figure 3:**
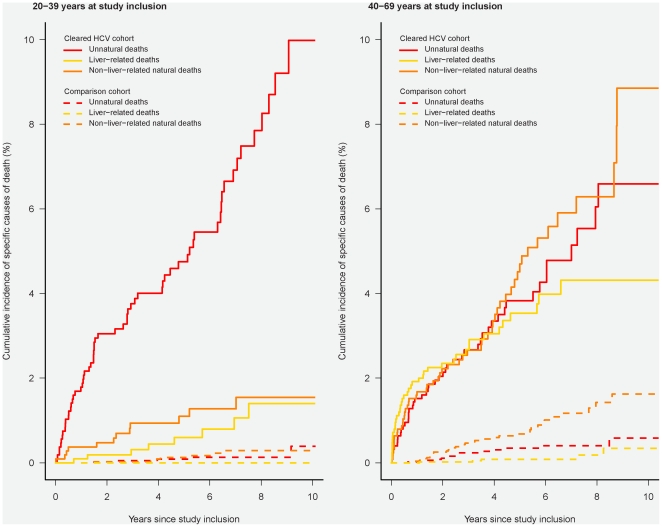
Cumulative incidence function illustrating cause-specific mortality in patients with cleared HCV-infection and an age- and gender-matched comparison cohort without comorbidity, alcohol abuse or IDU, according to age at study inclusion. Solid line: Patients with cleared HCV-infection; broken line: Individuals from the comparison cohort. The left-hand graph illustrates survival in individuals aged 20–39 years at study inclusion; the right-hand graph illustrates survival in those aged 40–69 years at inclusion.

**Figure 4:**
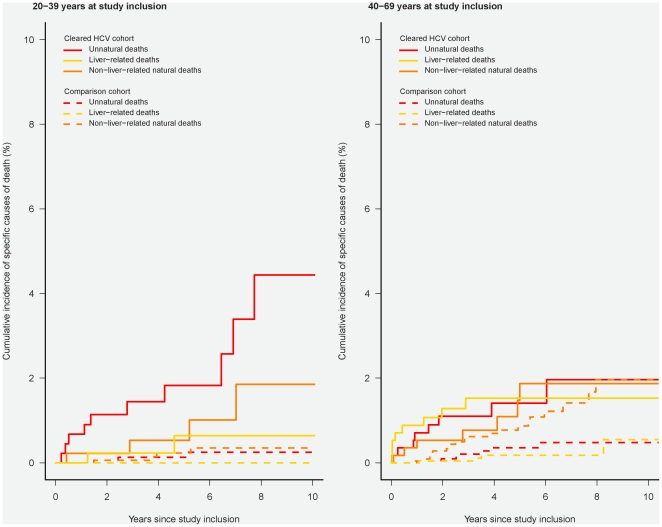
Cumulative incidence function illustrating cause-specific mortality in patients with cleared HCV-infection without comorbidity, alcohol abuse or IDU and an age- and gender-matched comparison cohort without comorbidity, alcohol abuse or IDU, according to age at study inclusion. Solid line: Patients with cleared HCV-infection; broken line: Individuals from the comparison cohort. Left- hand graph illustrates survival in individuals aged 20–39 years at study inclusion; right-hand graph illustrates survival in those aged 40–69 years at inclusion.

Specific causes of death are illustrated in Figure 3 and Appendix 4. In patients aged 20–39 years with cleared HCV-infection we observed 60 unnatural deaths with 32 of these being categorised as “accidental poisoning by and exposure to noxious substances” (ICD10: X40–X49). The 10-year risk of unnatural death was 10% (95% CI: 7%–13%), the 10-year risk of non-liver-related natural death was 2% (95% CI: 1%–3%) and the 10-year risk of liver-related death was 1% (95% CI: 1%–3%). Even in patients aged 20–39 without substance abuse or comorbidity, the 10-year risk of unnatural death was 4% (95% CI: 2%–9%) (Figure 4). In patients with cleared HCV-infection aged 40–69 years, the 10-year risk of unnatural death was 7% (95% CI: 5%–9%), the 10-year risk of non-liver-related natural death was 9% (95% CI: 6%–13%) and the 10-year risk of liver-related death was 4% (95% CI: 3%–6%).

## Discussion

In this nationwide cohort study, we demonstrated that patients with cleared HCV-infection suffer from a substantial risk of death, by far exceeding that of individuals of the same age and sex from the general population without comorbidity or substance abuse. However, some subgroups of patients have a reasonable prognosis, especially those aged 40–69 years without comorbidity or substance abuse. Their 10-year survival was similar to that of the comparison cohort. However, 50% of our study population had substance abuse, either alone or in combination with comorbidity, and they were at a substantially elevated risk of death; 10-year survival in patients aged 40–69 with substance abuse and comorbidity was approximately 49% (95% CI: 33%–74%).

### Strengths and limitations

The main limitation of our study was its reliance on national registries rather than clinical data to capture substance abuse and comorbidities. Expert opinion and our own clinical experience suggest that the majority of HCV-infected patients in Denmark are IDUs [26]. However, national registry data indicated that only 47% of patients were IDUs. Thus some of the excess mortality in patients aged 20–39 years who were not categorized as having drug abuse could, in part, be explained by misclassification of IDU. This suggestion is supported by the 4% 10-year risk of unnatural death in patients aged 20–39 years without documented substance abuse or comorbidity. Misclassification of IDU also may affect those aged 40–69 years; however, we suspect that these patients are more likely to be former IDUs, while younger patients affected by misclassification are more likely to be current IDUs. This difference could explain why patients aged 40–69 years without recorded substance abuse or comorbidity did not have any substantial increase in mortality, while those aged 20–39 years without recorded substance abuse or comorbidity were at increased risk of death. Another limitation of the registration of IDU is that it does not allow us to distinguish former from current users. Whereas the RDT in principle allows us to distinguish between IDUs currently undergoing treatment from IDUs previously having undergone treatment, it is impossible for us to determine whether previously treated patients are reusing drugs, and further we cannot preclude the possibility that IDUs currently undergoing treatment are in fact also using drugs. There were also limitations in our registration of alcohol abuse. We used the Danish National Hospital Registry and thereby we missed cases of alcohol abuse not leading to hospital contacts. Especially moderate alcohol abuse thereby might have been unrecognized. Further, we were unable to establish any dose-response relationship regarding alcohol abuse, as the ICD8- and 10 codes used could not be translated into any quantitative measure of alcohol abuse. Another study limitation is lack of data on antiviral therapy. However, as inclusion of patients depended on the first HCV RNA measurement following the first positive HCV-antibody test, we find it unlikely that a large proportion received antiviral therapy (as the natural sequence leading to treatment normally would be positive HCV antibodies followed by a positive HCV RNA test). Further, median year of inclusion was 2002 when treatment activity was still low in Denmark.

### Discussion of our results and the literature

Several cohort studies, including our own, have found increased mortality in HCV-infected patients [1]–[3]. This may be partly explained by the effects of the viral infection [13], [27]–[29] and partly by socioeconomic risk factors and risk-taking behaviours [3], [30]–[32]. Our study extends earlier findings by identifying a subset of patients with cleared HCV-infection, whose mortality is similar to that of an age- and gender- matched comparison cohort with no known risk factors (*i.e.*, those aged 40–69 years without recorded comorbidity or substance abuse). However, this subgroup accounted for only a quarter of patients with cleared HCV-infection; the remaining three-quarters had a substantially higher risk than the comparison cohort. Our data therefore suggest that serious attention should be paid to treating comorbidity and substance abuse, in addition to optimal medical treatment of the viral infection. Comorbidity and abuse had a substantial impact on mortality in patients aged 40–69 years with a 10-year survival of approximately 75% or below. This might be explained the high proportion of patients with severe comorbidity and liver diseases in this age group. In a Swiss cohort of 1,645 HCV-infected patients, Prasad *et al.* found that individuals without cirrhosis, HIV- or HBV-coinfection, alcohol abuse or IDU, who had a genotype other than genotype 3, were not at substantially increased risk of death compared to the general population (standardized mortality ratio = 1.14) [4]. However, as mortality in the general population might be driven in part by alcohol abuse, the lack of an association between HCV and mortality might be caused to some degree by bias introduced by the prevalence of alcohol abuse in the general population.

From a clinical vantage point, we extend the study by Prasad *et al.* by providing absolute risk estimates stratified by age and risk group, thus providing input for the clinical care of those who clear the virus. Consistent with previous findings for HCV-infected patients, individuals in our study had an increased risk of unnatural death [1], [3], especially in the younger age group, which is likely to stem from their risk-taking behaviours [30], [32]. Patients with cleared HCV-infection also had an increased risk of liver-related death. This could be explained by diagnostic bias in patients referred for evaluation of liver disease, which could have been caused by another agent (such as alcohol) and by evolution of an adverse cirrhotic course despite clearance of the virus. The finding of an increased risk of non-liver-related natural deaths in patients with cleared HCV-infection is probably due to the presence of comorbidity in this group.

We conclude that a minority of HCV-infected patients who clear the virus (those aged 40–69 with no comorbidity or substance abuse) has a mortality rate comparable to individuals without HCV (or comorbidity or substance abuse). However, most patients have an elevated mortality rate despite clearance of the virus. While viral clearance is an essential part of HCV management, our study highlights the importance of also focusing other conditions, such as alcohol abuse, IDU and comorbidity, which might increase mortality.

## Supporting Information

Appendix S1 **Definitions of alcohol abuse, IDU and HIV infection.**
(DOC)

Appendix S2 **ICD-10 codes for specific causes of death.**
(DOC)

Appendix S3 **Coding algorithm used for comorbid conditions in Charlson Comorbidity Index.**
(DOC)

Appendix S4 **Specific causes of death.**
(DOC)
