# Supplementary material for: Mortality among Patients with Cleared Hepatitis C Virus Infection Compared to the General Population: A Danish Nationwide Cohort Study
Source: PLoS One. 2011 Jul 18;6(7):e22476. doi: 10.1371/journal.pone.0022476 (PMC3138785; doi:10.1371/journal.pone.0022476)
Supplement: Appendix S1 — Definitions of alcohol abuse, IDU and HIV infection. (DOC) [file pone.0022476.s001.doc]

**Appendix 1:**  Definitions of alcohol abuse, IDU and HIV infection.

*Alcohol abuse*

A diagnosis in *the Danish National Hospital Registry* of ICD-8 codes: 291.00 - 291.99, 571.09, 571.10, 303.00 – 89, 303.91 – 99 and ICD-10 codes: K 70.0 – 70.9, F10.2 – 10.9, G31.2.

*Injection drug use*

Registration in *the* *Registry of Drug Abusers Undergoing Treatment* and/or a diagnosis in *the Danish National Hospital Registry* of ICD-8 codes: 304.09–304.99 and ICD-10 codes: F11.0–19.9, T40.0–40.9. Although *the* *Registry of Drug Abusers Undergoing Treatment* registers various kinds of substance abuse (injection drug use as well as other routes of drug use), we used all contacts to this registry to define IDU, as individuals with the combination of positive HCV antibodies and registration in this registry are very likely to be (former or current) injection drug users.

*HIV infection*

A diagnosis in *the Danish National Hospital Registry* of ICD-8 code: 07.983 and ICD-10 codes: B20.0-24.9.
