# Supplementary material for: Mortality among Patients with Cleared Hepatitis C Virus Infection Compared to the General Population: A Danish Nationwide Cohort Study
Source: PLoS One. 2011 Jul 18;6(7):e22476. doi: 10.1371/journal.pone.0022476 (PMC3138785; doi:10.1371/journal.pone.0022476)
Supplement: Appendix S2 — ICD-10 codes for specific causes of death. (DOC) [file pone.0022476.s002.doc]

**Appendix 2.** ICD-10 codes for specific causes of death.

The following definitions for specific causes of death were used according to the diagnosis listed as the primary cause of death (and corresponding ICD-10 codes) in the Danish Registry of Causes of Death.

Liver related deaths:

- Viral hepatitis (B15.0 – B19.9, B94.2)
- Primary liver cancer (C22.x).
- Alcoholic liver disease (K70.x).
- Non-alcoholic liver disease (K71.0 - K77.8).
- Oesophageal or gastric varices (I85.x, I86.4).

Non-liver related natural deaths:

- Death due to certain infectious and parasitic diseases (excluding viral hepatitis) (A00.0 – B14.9, B20.0 – B94.1, B94.3 – B99.9).
- Death due to neoplasms (excluding primary liver cancer) (C00.0 – C21.9, C23.0 - D49.9).
- Death due to endocrine, nutritional and metabolic diseases (E00.0 – E90.9).
- Death due to diseases of the nervous system (G00.0 – G99.9).
- Death due to diseases of the circulatory system (excluding oesophageal and gastric varices) (I00.0 – I83.9, I86.0- I86.3, I86.8- I99.9).
- Death due to diseases of the respiratory system (J00.0 – J99.9).
- Death due to diseases of the digestive system (excluding liver diseases) (K00.0 – K67.8, K80.0 – K93.8).
- Miscellaneous
  - Death due to diseases of the blood and blood-forming organs and certain disorders involving the immune mechanism (D50.0 – D89.9).
  - Death due to mental and behavioural disorders (F00.0 F09.9, F20.0– F99.9).
  - Death due to diseases of the skin and subcutaneous tissue (L00.0 – L99.9).
  - Death due to diseases of the musculoskeletal system and connective tissue (M00.0 – M99.9).
  - Death due to diseases of the genitourinary system (N00.0 – N99.9).
  - Death due to pregnancy, childbirth and the puerperium (O00.0 – O99.9)
  - Death due to symptoms, signs and abnormal clinical and laboratory findings, not elsewhere classified (R00.0 – R99.9).
  - Missing causes of death.

Unnatural deaths:

- Death due to mental and behavioural disorders due to psychoactive substance use (F10.0 – F19.9).
- Death by external causes (V00.0 – W99.9, Y00.0 – Y99.9).
