# Supplementary material for: Mortality among Patients with Cleared Hepatitis C Virus Infection Compared to the General Population: A Danish Nationwide Cohort Study
Source: PLoS One. 2011 Jul 18;6(7):e22476. doi: 10.1371/journal.pone.0022476 (PMC3138785; doi:10.1371/journal.pone.0022476)
Supplement: Appendix S3 — Coding algorithm used for comorbid conditions in Charlson Comorbidity Index. (DOC) [file pone.0022476.s003.doc]

**Appendix 3. Coding algorithm used for comorbid conditions in Charlson Comorbidity Index.**

| Comorbid condition in Charlson Comorbidity Index | ICD 8 | ICD 10 | Score |
| --- | --- | --- | --- |
| Myocardial infarction | 410 | I21;I22;I23 | 1 |
| Congestive heart failure | 427.09;427.10;427.11,427.19;428.99;782.49 | I50; I11.0; I13.0; I13.2 | 1 |
| Peripheral vascular disease | 440-445 | I70; I71; I72; I73; I74; I77 | 1 |
| Cerebrovascular disease | 430-438 | I60-I69; G45; G46 | 1 |
| Dementia | 290.09-290.19;293.09 | F00-F03; F05.1; G30 | 1 |
| Chronic pulmonary disease | 490-493;515-518 | J40-J47; J60-J67; J68.4; J70.1;J70.3; J84.1; J92.0; J96.1; J98.2; J98.3 | 1 |
| Connective tissue disease | 712;716;734;446;139.99 | M05; M06; M08; M09;M30- M36; D86 | 1 |
| Ulcer disease | 530.91; 530.98,531-534 | K22.1; K25-K28 | 1 |
| Diabetes mellitus type 1 and 2 | 249.00;249.06;249.07;249.09;250.00;250.06;250.07;250.09 | E10.0, E10.1; E10.9;E11.0; E11.1; E11.9 | 1 |
| Hemiplegia | 344 | G81; G82 | 2 |
| Moderate to severe renal disease | 403;404;580-584;590.09;593.19;753.10-753.19;792 | I12; I13; N00-N05; N07; N11; N14;N17-N19; Q61 | 2 |
| Diabetes mellitus type 1 and 2 with end organ damage | 249.01-249.05; 249.08; 250.01-250.05;250.08 | E10.2-E10.8;E11.2-E11.8 | 2 |
| Any tumor | 140-194 | C00-C75 | 2 |
| Leukemia | 204-207 | C91-C95 | 2 |
| Lymphoma | 200-203 | C81-C85; C88; C90; C96 | 2 |
| Metastatic solid tumor | 195-199 | C76-C80 | 6 |
| AIDS * | 79.83 | B21-B24 | 6 |
| Mild liver disease ** | 571; 573.01; 573.04 | B18; K70.0-K70.3; K70.9; K71; K73;K74; K76.0 | 1 |
| Moderate to severe liver disease ** | 70.00;70.02;70.04;70.06;70.08;573.00;456 | B15.0; B16.0; B16.2; B19.0; K70.4;K72; K76.6; I85 | 3 |

*: Patients with HIV were excluded from the study.

**: The liver diseases of the CCI were not included in our comorbidity score, as they were considered being part of the causal pathway of mortality.
