# Supplementary material for: Mortality among Patients with Cleared Hepatitis C Virus Infection Compared to the General Population: A Danish Nationwide Cohort Study
Source: PLoS One. 2011 Jul 18;6(7):e22476. doi: 10.1371/journal.pone.0022476 (PMC3138785; doi:10.1371/journal.pone.0022476)
Supplement: Appendix S4 — Specific causes of death. (DOC) [file pone.0022476.s004.doc]

**Appendix 4.** Specific causes of death.

|  |  | **Total cohort** | **20 – 39 years at study inclusion** | **40 – 69 years at study inclusion** |
| --- | --- | --- | --- | --- |
| **Liver related deaths** |  |  |  |  |
| b16 | Acute hepatitis B | 1 | 0 | 1 |
| b18 | Chronic viral hepatitis | 6 | 2 | 4 |
| c22 | Malignant neoplasm of liver and intrahepatic bile ducts | 3 | 0 | 3 |
| k70 | Alcoholic liver disease | 35 | 5 | 30 |
| k74 | Fibrosis and cirrhosis of liver | 4 | 1 | 3 |
| **Natural deaths** |  |  |  |  |
| a00 - b19 and b25 - b99 | Certain infectious and parasitic diseases | 2 | 0 | 2 |
| b20 - b24 | Human immunodeficiency virus [HIV] disease | 1 | 1 | 0 |
| c00 - d48 | neoplasms | 19 | 1 | 18 |
| e00 - e90 | Endocrine, nutritional and metabolic diseases | 5 | 1 | 4 |
| g00 - g99 | Diseases of the nervous system | 3 | 1 | 2 |
| i00 - i99 | Diseases of the circulatory system | 12 | 5 | 7 |
| j00 - j99 | Diseases of the respiratory system | 6 | 0 | 6 |
| k00 - k93 | Diseases of the digestive system | 7 | 1 | 6 |
| m00 - m99 | Diseases of the musculoskeletal system and connective tissue | 1 | 1 | 0 |
| missing |  | 2 | 0 | 2 |
| n00 - n99 | Diseases of the genitourinary system | 1 | 0 | 1 |
| q00 - q99 | Congenital malformations, deformations and chromosomal abnormalities | 1 | 0 | 1 |
| r00 - r99 | Symptoms, signs and abnormal clinical and laboratory findings, not elsewhere classified | 5 | 1 | 4 |
| **Unnatural deaths** |  |  |  |  |
| f10-f19 | Mental and behavioural disorders due to psychoactive substance use | 27 | 8 | 19 |
| x40 - x49 | Accidental poisoning by and exposure to noxious substances | 44 | 32 | 12 |
| X60-X84 | Intentional self-harm | 10 | 7 | 3 |
| X85-Y09 | Assault | 2 | 2 | 0 |
| y10-y34 | Event of undetermined intent | 22 | 11 | 11 |
